# Sex Differences in Physical Attractiveness Investments: Overlooked Side of Masculinity

**DOI:** 10.3390/ijerph19073842

**Published:** 2022-03-24

**Authors:** Marta Kowal, Piotr Sorokowski

**Affiliations:** Institute of Psychology, University of Wrocław, 50-529 Wrocław, Poland; piotr.sorokowski@uwr.edu.pl

**Keywords:** gender, diary study, enhancing beauty, self-modification, sex comparison

## Abstract

Background: Public opinion on who performs more beauty-enhancing behaviors (men or women) seems unanimous. Women are often depicted as primarily interested in how they look, opposed to men, who are presumably less focused on their appearance. However, previous studies might have overlooked how masculinity relates to self-modification among men. Methods: We explored this issue in depth by conducting a qualitative Study 1 aimed to establish how men and women enhance their attractiveness (*N* = 121) and a quantitative Study 2 aimed to test time spent on activities that increase one’s attractiveness in a longitudinal design (with seven repeated measures from 62 participants; *N_(total)_* = 367). Results: We observed no sex differences in beauty investments. Although women spent more time on make-up and cosmetics usage, men caught up with women in exercising and bodybuilding. Conclusion: Our study provides evidence that there may not be such wide sex differences in the intensity of enhancing one’s appearance as has been previously thought. We hypothesize that this might partly stem from changes in gender roles regarding masculinity.

## 1. Introduction

Pursuing a good look is a widely observed phenomenon [1]. The attraction to attractiveness seems to be deeply rooted in our nature. Langlois et al. [2] showed that 2–8-month infants look longer at attractive faces (compared to unattractive). Further investigations found this preference among newborns even as young as 14 h from birth [3]. Numerous studies provided evidence that attractiveness ratings are based on, for instance, masculinity level [4], facial features (skin tone: [5]; facial hair: [6]; hair color: [7]), or clothing [8,9]. Each of those bodily characteristics can be, to some extent, altered. For instance, skin tone can be modified with makeup; facial hair—trimmed; hair color—changed; clothing—chosen in a more fitting fashion.

Drawing on a myriad of studies on the benefits of physical attractiveness (see [10]), it seems that everyone should be interested in improving their appearance. Interestingly, previous studies provided evidence that women exceed men in time and energy spent enhancing the way they look [11]. Although many cosmetic companies are conducting large-scale advertising campaigns, encouraging men to buy ‘male cosmetics products’, it is still women who spend more money on cosmetics ([12], but see [13] for contradictory evidence from India). It might be connected to the fact that using cosmetics and care products is assigned as a typically female and not male activity [14]. However, it does not necessarily mean that men do not enhance their attractiveness—they may engage in different activities to increase their appeal; for instance: muscle building.

Some scholars suggested that modern culture encourages men to care and invest in their appearance [15]. With an increasing share of male’s body displays in media over the last few decades [16], it seems understandable that men are becoming more and more aware of how their appearance is perceived. An increased body awareness often entails a willingness to engage in self-modification [17,18]. These changes might be a repercussion of the alleged masculinity crisis [19,20]. This is a crisis that has influenced social expectations of men who report feeling pressure to attain (and maintain) a muscular body that would fit the one cultivated by media beauty standards [21,22]. This converges with evolutionary theories, which highlight the historical importance of male upper body strength in intra- and inter-sexual competition [23]. For instance, women rate muscular (vs. non-muscular) male bodies more favorably [4]. That may explain why men are more interested in their muscularity than women (see [24,25]).

However, it is crucial to first establish how exactly women and men attend their attractiveness and second, how much time they spend improving their look. Unfortunately, to date, most of the studies focused mainly on cosmetics usage or plastic surgeries when investigating beauty-enhancing behaviors, overlooking different arrays of appearance improving activities among men and women [11]; or limited their investigations to asking single retrospective questions [14,26].

To fill this knowledge gap, we conducted two studies: a qualitative study, in which we established how people improve their appearance, and second, a week-long diary study, in which we compared the intensity of beauty-enhancing behaviors among men and women daily. We predicted that men and women enhance their attractiveness in different ways (Hypothesis 1) and that women exceed men in time spent enhancing their attractiveness (Hypothesis 2). Noteworthy, people tend to compare themselves with images of models from mass media, who set (unrealistic) beauty standards [27]. Those who fail to reduce discrepancies between their bodies and perfect social media bodies report lower self-esteem and negative self-perceived attractiveness [28]. Conversely, those who actively engage in achieving a better look, report higher self-attractiveness [29]. Thus, we expected more beauty-enhancing activities to be performed by individuals who consider themselves more attractive (Hypothesis 3). Furthermore, we controlled for narcissism because more narcissistic individuals tend to focus on their looks [30,31]. Therefore, we predicted that those high in narcissism would spend more time improving their look (Hypothesis 4).

## 2. Materials and Methods

### 2.1. Study 1

We posted invitations to participate in a short online study on attractiveness on various social media groups. The invitation was accepted by 121 individuals (60% women). Participants’ age ranged from 18 to 49 (*M* = 24.75, *SD* = 6.59). Participants were encouraged to provide as many answers as possible to two open-ended questions: ‘what do people do to improve their physical attractiveness?’ and ‘how do you enhance the way you look?’. Recurring answers were narrowed to establish a list of the most common beauty-enhancing behaviors. The final list included eight types of behaviors (in brackets are the percentages of participants who mentioned the given activity): make-up usage (mentioned by 59% of respondents), cosmetics usage (58%), cardio exercises (57%), strength exercises (55%), hair grooming (35%), body cleaning (34%), hands grooming (22%), and looking in the mirror and adjusting one’s image (17%).

### 2.2. Study 2

Prior to Study 2, we ran a simulation with 5000 iterations to determine the sample size needed to achieve a power of at least 0.8. The power analysis revealed that a sample of 60 participants with seven repeated measures in a multilevel model is needed to detect a medium effect size (fixed effect of 0.3, power 0.82, 95% CI [79.90, 84.71], alpha 0.05). To account for potential missing data, we decided to recruit 62 participants. Participants were asked to fill an online diary each day for a week, reporting how much time they spent enhancing their beauty each day.

#### 2.2.1. Participants

We posted direct and indirect (i.e., directed towards relatives of social media users) invitations to participate in a week-long study on various social media groups. Sixty-two participants (58% women) agreed to participate, among whom 33 kept the diary and provided answers on each of the seven days (*N_total_* = 367). For the whole sample, the mean of daily diary entries (i.e., the number of days a participant filled a diary) was 3.76 (*SD* = 1.99). Participants’ age ranged from 20 to 67 (*M* = 31.74, *SD* = 10.72). Noteworthy, men and women did not differ in terms of their age (*t*_(62)_ = 0.27, *p* = 0.79) and education (*t*_(62)_ = −0.56, *p* = 0.58). All participants provided informed written consent to take part in the study. The ethical approval for conducting the study was granted by the Institutional Review Board at the Institute of Psychology, University of Wrocław (IPE0022, 2020); the study has been performed following the ethical standards laid down in the 1964 Declaration of Helsinki and its later amendments.

#### 2.2.2. Measures and Procedure

Participants were reminded each evening over seven days to keep an online diary. Participants provided basic socio-economic information on the first day of the study (i.e., age, sex, education status). They filled the Narcissistic Personality Inventory–13 [32], self-perceived physical attractiveness measure, and a daily intensity of enhancing one’s physical attractiveness. Participants reported time spent improving their attractiveness and self-perceived attractiveness on all subsequent days.

The Narcissistic Personality Inventory–13 [32] is a widely recognized [33] 13-item questionnaire (Polish adaptation: [34]) to measure the narcissism trait. Each item consists of two opposite statements, and participants choose the one that they most agree with. Exemplary item’s statements include: ‘When people compliment me, I get embarrassed’; ‘I know that I am a good person because everybody keeps telling me so.’ The scale was acceptably [35] reliable (Cronbach’s alpha: 0.64). Self-perceived physical attractiveness measure consists of three questions: ‘how attractive would you rate yourself?’; ‘how would other women rate your attractiveness?’; ‘how would other men rate your attractiveness,’ with answers ranging from 1—very unattractive to 7—very attractive (Cronbach’s alpha: 0.92). An appearance-improving scale consisted of eight questions regarding the time spent performing eight types of activities (i.e., make-up usage, hands grooming, cosmetics usage, body cleaning, hair grooming, strength exercises, cardio exercises, and looking in the mirror and adjusting one’s image). Participants used a slider to indicate the number of minutes spent performing a given activity during each day (ranging from one minute to six hours or more).

#### 2.2.3. Statistical Analysis

To obtain a composite score of the Narcissistic Personality Inventory–13, we computed a sum of all the items (range: 1–13). Further, to obtain a measure of self-perceived physical attractiveness, we calculated a daily mean score of attractiveness (ranging from 1 to 7). Then, we computed two composite scores of everyday physical attractiveness-enhancing behaviors: first, including the sum of minutes spent performing six out of eight beauty improving behaviors (i.e., make-up usage, hands grooming, cosmetics usage, body cleaning, hair grooming, and looking in the mirror and adjusting one’s appearance), and the second, including all eight behaviors (with the addition of strength and cardio exercises).

In the next step, we tested two linear mixed models. In these models, responses were nested within participants. In the first model, we regressed a daily measure of the intensity of physical attractiveness-enhancing behaviors (without physical exercises) on participants’ sex, age, relationship status (either single or in an intimate relationship), narcissism score, and self-perceived physical attractiveness. The second model differed as the outcome variable also included time spent on physical exercises. Both models included a random intercept of the daily intensity of appearance-enhancing behaviors on a participant level. All the statistical analyses were performed in Jamovi (Version 1.2.16.) (Jamovi Project, Sydney, Australia).

## 3. Results

**Figure 1:**
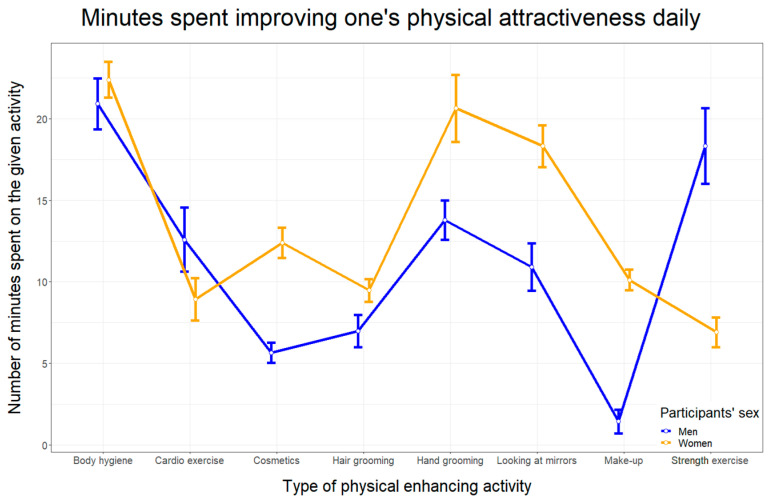
Means and standard errors of daily time spent performing each beauty-enhancing activity regarding participants’ sex.

Figure 1 shows the mean number of minutes participants spent daily on each physical enhancing activity concerning sex.

Table 1 presents a summary of participants’ beauty-enhancing behaviors (in minutes per day), along with narcissism and self-perceived attractiveness scores.

As illustrated in Table 2, the only difference between the two models is that sex was significantly related to the intensity of daily performed behaviors aimed at increasing one’s attractiveness when not considering the time spent exercising. However, sex was not significant when including the time spent exercising. In both models, self-perceived attractiveness was positively related to the amount of physical enhancing behaviors, while the relationship status was negatively related to the intensity of improving one’s look. Age and narcissism scores were not associated with beauty-enhancing activities. We also checked for potential outliers that might have driven the observed relationships. To this goal, we calculated Mahalanobis Distance and relied on the usually recommended cut-off (i.e., *p* < 0.001; [36,37]) when screening for potential outliers. We identified 19 data points as potential outliers. However, running the analyses with and without them yielded the same pattern of results, so we retained the entire dataset.

## 4. Discussion

The present study aimed to compare the intensity of beauty-enhancing behaviors among men and women. Our results provide evidence that ways to achieve a good look vary between both sexes (H1), but, more importantly, both women and men may spend relatively the same amount of time improving their appearance (H2). We found that women exceeded men in using cosmetics and putting on make-up, while men more intensively altered their bodies via exercises. In addition, self-perceived attractiveness (H3) and being in a relationship were positively related to the time spent enhancing beauty, while narcissism (H4) and age were non-related.

Corroborating results of previous studies [11,38] and in line with Hypothesis 1, women spent more time on cosmetics and make-up usage than men. The cosmetics industry mainly targets women to become consumers [39]; but see [40] for evidence of increasing popularity of male care products). Mate market perspective offers an explanation of this phenomenon [41] and refers to different adaptive challenges that men and women face. Men are to find a healthy and fertile partner with whom they could have offspring (while women are to find men of high status who could provide for them and their offspring). Many health and fertility indicators are situated on the face (clear and smooth skin, large eyes with a limbal ring, skin tone; [5,42]), which might be why facial attractiveness is often considered as one of the most important aspects of female beauty [43]. Notably, cosmetics and make-up provide an excellent way to enhance female facial attractiveness [44], which women eagerly take advantage of [14].

On the other hand, men’s way of reaching a high status and interest from women may be through attaining a muscular body [45]. Muscularity and masculinity were found to be positively associated with formidability and strength, often translating into enhanced social status [46]. More muscular men are rated as more attractive by both men [47] and women [48]. Altogether, it may explain why we observed that men spent more time on exercises than women, supporting Hypothesis 1 and previous findings [49].

Contrary to a common opinion that women “[spend] hours (…) each week on makeup, hair styling and curating an outfit”, while looking attractive for men “often just mean[s] business casual clothing and a short haircut” [50], we did not find support for Hypothesis 2. It is noteworthy that traditional gender roles seem to have been changing in recent years [51]. Although grooming was traditionally considered a typically female activity [15], it also has become accepted (and sometimes even expected; [22]) from men [20,21]. Presumably, the alleged sex grooming gap is not so wide as previously thought [14]. Future studies should include measures of traditional female and male roles to test the extent to which gender roles could drive beauty-enhancing behaviors.

Considering a common agreement on the advantages of being perceived as beautiful and disadvantages of being perceived as less attractive [52], one could expect that intensified activities aimed at improving one’s appearance should be displayed especially by people who acknowledge the shortcomings of their looks. Those convinced about their visual appeal would not need to devote additional energy to improving their already beautiful appearance [53]. Some initial evidence for such patterns was provided by a study on Lebanese women [54], which showed that those with higher self-esteem used fewer cosmetics than those with lower self-esteem.

However, other studies provided contradictory evidence [29]. For instance, individuals who have undergone plastic surgery reported being more attractive and satisfied with their appearance [55]. Additionally, others rated post-surgery images as more attractive [56], which could potentially act as a positive strengthening force for those who go to great lengths to look beautiful. Our study supports a positive relationship between ratings of self-attractiveness and time spent enhancing one’s look (Hypothesis 3). Several theoretical frameworks (such as body objectification [57] and cultural approach [58]) provide an explanation for these observations. Internalizing widespread beauty ideals from mass media results in higher body awareness and treating one’s body as an object malleable to modifications [59]. Thus, those who do not actively engage in increasing one’s appearance might notice a wider gap between their look and the look of social media models. However, due to the observational nature of our study, we cannot derive whether the belief of one’s attractiveness stems from intensified self-care, or instead, being beautiful leads to increased efforts in preserving one’s beautifulness.

Moreover, we found no evidence for Hypothesis 4, as there was no relationship between the narcissism score and the intensity of beauty-enhancing behaviors. It may seem surprising, as narcissistic individuals are highly concerned with their look [30]. For example, Sorokowski et al. [31] found links between narcissism and the frequency of posting images of one’s appearance on social media (i.e., selfies). However, the present results suggest that preoccupation with one’s look among narcissistic people might not necessarily entail intensified activities to increase one’s attractiveness. Notably, the effect size of the relationship between the intensity of beauty investments and narcissism might have been too small to detect in the present study. Additionally, the reliability score of the scale limits drawing more general conclusions. Thus, more research is needed to disentangle the relationship between beauty-enhancing behaviors and narcissism.

We also found that being in a romantic relationship is negatively related to the amount of time spent improving one’s look. Singles spent more time enhancing their appearance than individuals in a relationship. It seems reasonable, as single individuals face the challenge of finding a potential partner while individuals in a relationship already have one. In addition, having a partner is related to numerous advantages, such as decreased levels of stress [60], increased levels of happiness [61], and more intense feelings of being loved [62]. Thus, people in a relationship might feel accepted for who they are more than how they look [63], which leads to a decreased propensity to engage in physical attractiveness-related activities.

Although the present study contributes to a better understanding of beauty-enhancing behaviors and their predictors, it is not without limitations. First, the sample size and the recruitment method (i.e., via social media and social media users’ relatives) limits drawing general conclusions. More research with larger and diverse samples (in terms of socio-economic background) with a longitudinal design (over a more extended time) is needed to confirm the present results. It could also be illuminating to test for any cross-cultural differences in the studied phenomena. Moreover, the intensity of activities aimed at improving one’s look was self-reported. Thus, the exact times might have been, to some degree, biased.

## 5. Conclusions

To sum up, our study highlights the need to treat activities aimed at improving physical attractiveness more broadly. Enhancing one’s look ceases to be merely about cosmetics or make-up usage [64]; it expands into all behaviors that both men and women perform to increase their appeal in the eye of others. Even though our results show that women spend more time on make-up and cosmetics usage, men catch up with women exercising and building their muscles. It may relate to different gender roles, including masculinity being related to muscularity and femininity being associated with make-up. Furthermore, we found that individuals who feel more attractive spend more time improving their attractiveness. However, more studies are needed to discover whether beliefs of one’s beauty are caused by more extensive beauty-enhancing behaviors or instead being beautiful (and experiencing social benefits) prompts people to perform more activities to preserve the good look and to disentangle the interrelationship between gender roles and beauty-enhancing activities.

## Figures and Tables

**Table 1:** A summary of participants’ characteristics.

Variable	Men (*N* = 26)		Women (*N* = 36)	
	Mean (*SD*)/Median	Range	Mean (*SD*)/Median	Range
Age	31.30 (8.99)/28.50	21–47	32.06 (11.93)/26.50	20–67
Narcissism (1–13)	3.81 (2.43)/3	0–8	3.31 (2.34)/3	0–8
Self-perceived physical attractiveness (1–7)	3.35 (1.18)/3.33	0–6	3.75 (1.01)/3.67	0.67–6
Make-up ^a^	1.44 (9.02)/0	0–105	10.12 (9.39)/10	0–40
Cosmetics ^a^	5.67 (7.61)/4	0–63	12.40 (13.63)/8	0–101
Hand grooming ^a^	13.78 (14.82)/8	0–69	20.63 (30.15)/14	2–356
Body hygiene ^a^	20.91 (19.32)/15	0–134	22.39 (16.04)/19	0–137
Hair grooming ^a^	6.98 (12.13)/4	0–99	9.48 (10.27)/7	0–90
Looking at mirrors and adjusting one’s image ^a^	10.91 (18.01)/7	0–171	18.32 (18.88)/11	0–94
Strength exercise ^a^	18.34 (28.51)/0	0–102	6.91 (14.29)/0	0–79
Cardio exercise ^a^	12.59 (24.13)/0	0–153	8.93 (19.10)/0	0–92

Note ^a^—in minutes.

**Table 2:** Results of multilevel models, with daily diary entries nested within participants and random effects for intercepts.

Model 1 (without Exercises)				Model 2 (with Exercises)		
Predictor	*β*	*SE*	*p*	*β*	*SE*	*p*
Sex	0.213	0.094	0.027 *	0.063	0.093	0.501
Relationship status	−0.588	0.200	0.005 **	−0.572	0.197	0.005 **
Narcissism	−0.067	0.105	0.529	−0.075	0.104	0.475
Self-perceived physical attractiveness	0.337	0.082	<0.001 ***	0.403	0.084	<0.001 ***
Age	0.080	0.096	0.409	0.011	0.095	0.905

Note * *p* < 0.05, ** *p* < 0.01, *** *p* < 0.001. Random effects of the first and second model for the intercept: SD = 0.599, ICC = 0.419 and LRT = 94.194, *p* < 0.001, and SD = 0.576, ICC = 0.376 and LRT = 81.922, *p* < 0.001, respectively.

## Data Availability

Data is accessible via the public repository under the link: https://figshare.com/s/1ec34e9c6af703cc8a8d (accessed on 7 February 2022).
